# Emergence of NDM-1-producing *Raoultella ornithinolytica* from reservoir water in Northeast Thailand

**DOI:** 10.14202/vetworld.2023.2321-2328

**Published:** 2023-11-27

**Authors:** Chutima Karnmongkol, Piyachat Wiriyaampaiwong, Mullika Teerakul, Jukkarin Treeinthong, Nattapong Srisamoot, Anupong Tankrathok

**Affiliations:** 1 Department of Biotechnology, Faculty of Agricultural Technology, Kalasin University, Kalasin, Thailand; 2 Department of Fisheries Technology, Faculty of Agricultural Technology, Kalasin University, Kalasin, Thailand; 3 Protein and Proteomics Research Center for Commercial and Industrial Purposes (ProCCI), Faculty of Science, Khon Kaen University, Khon Kaen, Thailand

**Keywords:** antibiotic resistance, carbapenemase, *Raoultella ornithinolytica*, waterborne pathogens

## Abstract

**Background and Aim:**

Antibiotic resistance is a major global health threat. The increasing prevalence of drug-resistant bacteria poses a serious challenge to the effective treatment of infections in both humans and animals. Water is a major source of human and animal exposure to bacteria, and the presence of drug-resistant bacteria in water could present a severe threat to public health and animal production. This study investigated the presence of drug-resistant bacteria in Lam Pao Dam (LPD) water in Kalasin, Thailand.

**Materials and Methods:**

Ampicillin-resistant strains were obtained from LPD water and identified using 16s rDNA sequencing. Antibiotic resistance genes were detected by polymerase chain reaction using specific primers. The presence of antibiotic-resistant bacteria was evaluated using 16s amplicon analysis. The minimum inhibitory concentration (MIC) of *Raoultella ornithinolytica* strains against antibiotics was determined.

**Results:**

A total of 12 *R. ornithinolytica*, 4 *Bacillus cereus*, and 4 *Enterococcus faecalis* isolates were resistant to ampicillin. Almost all *R. ornithinolytica* strains harbored *bla*_SHV_ and *bla*_OXA_ genes, and two strains also harbored the *bla*_NDM-1_ gene. All four *E. faecalis* strains harbored the *bla*_IMP_ gene. The most abundant species in the LPD sample was *Exiguobacterium indicum*, followed by *E. faecalis* and *R. ornithinolytica*. The MICs of 10 *R. ornithinolytica* strains against five antibiotics revealed that all strains were resistant to ampicillin but susceptible to meropenem, doripenem, ertapenem, and imipenem.

**Conclusion:**

These findings suggest a high prevalence of drug-resistant bacteria in LPD water. This is a cause for concern, as it could spread antibiotic-resistant infections in the community.

## Introduction

Antimicrobial resistance (AMR) in the environment poses a significant threat to both human health and ecosystem, resulting in biodiversity loss, ecological damage, and adverse effects on beneficial microbes. Recent reports from multiple countries have confirmed the presence of AMR in waterborne pathogens, emphasizing the significance of investigating antibiotic use and the impact of AMR on aquatic animals and the environment [1–5]. Hence, it is necessary to implement global strategies for eco-friendly development, improving the quality of life, and managing water resources to effectively address this increasing threat. Raising awareness and understanding of AMR across society are pivotal to achieving environmental quality and protecting human health. Drug-resistant bacteria can also exert a significant impact on animal production by causing infections in livestock, which can result in decreased productivity, increased mortality, and the need for expensive treatment. In some cases, drug-resistant infections can even result in the culling of entire herds of animals [6].

Carbapenems, life-saving antibiotics, have been considered the most potent drugs for treating multidrug-resistant Gram-negative bacteria [7]. Nevertheless, the worldwide prevalence of carbapenem-resistant *Enterobacteriaceae* (CRE) indicates that this antibiotic might become less effective in the future. Carbapenem-resistant *Enterobacteriaceae* becomes resistant to β-lactam antibiotics primarily through three mechanisms, namely carbapenemase production, efflux pumps, and porin mutations [8]. Therefore, it is important to increase the use of a limited number of last-resort antibiotics, such as polymyxins, fosfomycin, tigecycline, and rifampin, for the treatment of CRE infections [9].

*Raoultella ornithinolytica* is an encapsulated Gram-negative, non-motile, rod-shaped bacterium belonging to the *Enterobacteriaceae* family. *Raoultella* *ornithinolytica* was previously known as a relatively harmless pathogen that generally exists in aquatic environments and soil. However, extensive data have indicated an association between this emerging bacterium and severe clinical human infections such as gastrointestinal, hepatobiliary, and urological infections [10]. *Raoultella ornithinolytica* was recently recognized as a global emerging zoonotic pathogen of concern [11], and its presence has been identified in meat products [12]. Furthermore, *R. ornithinolytica* strains that produce extended-spectrum beta-lactamase (ESBL) have been reported, such as *Klebsiella pneumoniae* carbapenemase, New Delhi, metallo-β-lactamase (NDM), imipenem-resistant *Pseudomonas*-type carbapenemases (IMP), and oxacillinase (OXA-48) [13, 14].

**Figure-1:**
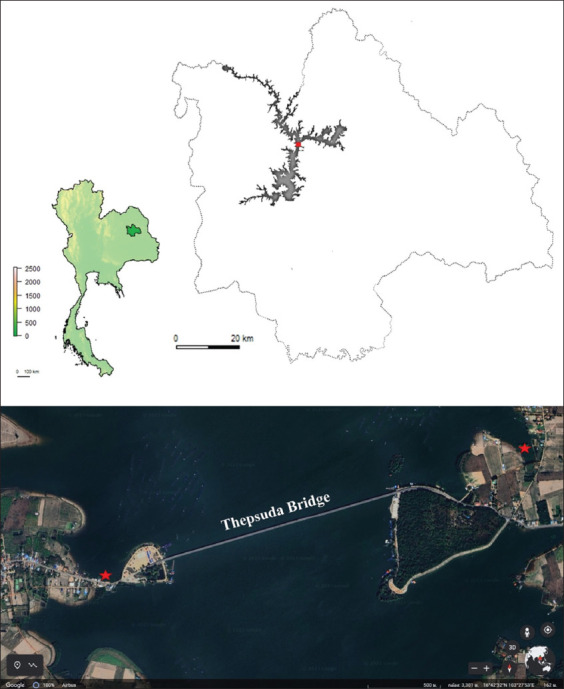
A map of LPD in Kalasin, Thailand. The LPD map was generated by modifying the Kalasin map from the GADM version 2.8 (https://gadm.org/maps.html), and the Thepsuda bridge view was illustrated using Google Earth (https://www.google.com/earth/index.html). Red stars indicate the sample collection sites. LPD: Lam Pao Dam

The Lam Pao Dam (LPD) in Kalasin Province, Thailand, is a large clay dam constructed in 1963. It serves as a flood control measure and an irrigation water source, with a height of 33 m and a length of 7.8 km (Figure-1). The dam has a storage capacity of 1980 million cubic meters of water (https://www.rid.go.th). It is an important freshwater fisheries resource, including a tilapia cage culture, and a popular tourist destination for swimming, boating, and fishing. Hence, LPD is valuable to the people of Kalasin province. Therefore, this study aimed to monitor the presence of AMR in LPD. We isolated and characterized microorganisms exhibiting AMR from the side-edge surface water samples of LPD.

## Materials and Methods

### Ethical approval

This study does not require ethical approval. This experiment does not contain any studies with human participants or animals performed by any of the authors.

### Study period and location

The study was conducted from February 2020 to December 2022 at LPD reservoir and the Department of Biotechnology, Faculty of Agricultural Technology, Kalasin University, Kalasin Province, Thailand.

### Isolation of resistant bacteria

Surface water samples of LPD were collected in February 2020 (Figure-1), and from each sample, 1.5 L was filtered using a 0.2-micron sterile filter. The filter paper was cultured in Luria–Bertani (LB) broth containing 50 mg/mL of ampicillin for 18 h at 37°C. Then, 10-fold serial dilutions of the cultured samples were prepared using sterile water as a diluent and the spread plate technique on different agar media such as thiosulfate–citrate–bile salts–sucrose agar (TCBS agar), *Salmonella*-*Shigella* agar (SS agar), blood agar, *Aeromonas* agar, and *Streptococcus* selection agar (S agar). The plate was then incubated for 18 h at 37°C. Different colonies on each plate were isolated into a pure colony, and then, the morphological characteristics, such as size, shape, and colony morphology, were observed. DNA was extracted from the isolated bacteria, and 16s rDNA was amplified by polymerase chain reaction (PCR) using appropriate primers. Next, the PCR products were sequenced (Macrogen, Seoul, South Korea), and the obtained nucleotide sequences were matched against nucleotide sequences present in GenBank using the BLAST program (https://blast.ncbi.nlm.nih.gov/blast.cgi). Phylogenetic trees were constructed using MEGA version X [15] and the neighbor-joining method.

### Detection of resistance genes

The isolated strains were cultured in LB broth for 18 h at 37°C and then collected for bacterial plasmid extraction, which was performed using the GF-1 Plasmid DNA Extraction Kit (Vivantis, Selangor DE, Malaysia) according to the manufacturer’s instructions. Then, the plasmid DNA extracts were quantitated using a spectrophotometer. The plasmid DNA template was applied to the PCR system to examine three beta-lactamase genes (*bla*_SHV_, *bla*_TEM_, and *bla*_OXA_) [16] and three carbapenemase genes (*bla*_NDM-1_, *bla*_KPC-2_, and *bla*_IMP_) [17], all of which are commonly found in CRE. The PCR was performed on a T100 Thermal Cycler (Bio-Rad, Hercules, CA, USA), and the products were visualized using 1% agarose gel electrophoresis.

### Microbiome analysis

The above-described filter paper-cultured media were used for microbial DNA extraction using the GF-1 bacterial DNA extraction kit (Vivantis) according to the manufacturer’s protocol. The 16s amplicon analysis was conducted by Novogene (Beijing, China). The v3–v4 region of 16s rRNA was amplified using 341F/806R primers and Phusion High-Fidelity PCR Master Mix (New England Biolabs), and the amplicons were purified using the Qiagen Gel Extraction Kit (Qiagen, Germany). Before the Illumina platform analysis, the amplicon library was generated using the NEBNext Ultra™ DNA Library Prep Kit (New England Biolabs, England) for Illumina and quantified using Qubit and Q-PCR. For obtaining clean data, the raw sequencing data were processed by merging and filtering using FLASH V 1.2.7 (http://ccb.jhu.edu/software/FLASH/) and QIME V 1.7.0 (http://qiime.org/scripts/split_libraries_fastq.html), respectively. Operational taxonomic units were clustered using the Uparse software (http://www.drive5.com/uparse/) based on a ≥97% sequence similarity threshold to visualize the composition of the microbial community. Species annotation was demonstrated using the Mothur software (https://mothur.org/) against the SSUrRNA database of the SILVA database.

### Drug susceptibility testing

Drug susceptibility assays were conducted using the resazurin-based 96-well plate microdilution technique applied in a previous study by Tankrathok *et al*. [18]. Briefly, the bacterial isolates were grown in Mueller Hinton Broth (MHB) at 37°C until they reached the middle log phase. Then, the cells were diluted to 10^4^ colony-forming unit/mL in phosphate-buffered saline (PBS, pH 7.2). Next, 25 μL of diluted cultured cells was mixed with 25 μL of twofold serial dilution antibiotics (ampicillin, meropenem, doripenem, ertapenem, and imipenem) (8, 4, 2, 1, 0.5, 0.25, 0.125, and 0.0625 μg/mL) in 96-well plates, followed by incubation for 3 h at 37°C. Before culturing at 37°C for 18 h, 50 μL of MHB was pipetted into the testing cell mixtures. Then, 30 μL of 0.015% w/v resazurin solution was added, and the mixture was placed at 37°C for 2 h to monitor the minimum inhibitory concentration (MIC), wherein the lowest antibiotic concentration presented a clear blue color.

## Results

### Isolation of drug-resistant bacteria

The resistant bacteria isolated from LPD water were processed by previously culturing in LB broth containing ampicillin and further growing on differential agar media. Differences in colony characteristics were picked and restreaked for single-type colonies. At least 20 ampicillin-resistant isolates were obtained, including 5 isolates from both *Aeromonas* agar and blood agar, 4 isolates from SS agar and S agar, and 2 isolates from TCBS agar. The 16s rDNA of each isolate was amplified and sequenced to identify the isolated bacterial species. Results showed that the resistant bacteria isolated from LPD water consisted of 12 *R. ornithinolytica*, 4 *Bacillus cereus*, and 4 *Enterococcus faecalis* that were obtained with identity percentages ranging from 99% to 100% (Table-1).

**Table-1:** 16s rDNA identification of bacterial strains isolated from LPD.

Isolates	Accession No.	GenBank database	Identity (%)
LPDA1	MW148492	*R. ornithinolytica*	99
LPDA2	MW148493	*R. ornithinolytica*	99
LPDA3	MW148494	*R. ornithinolytica*	99
LPDA4	MW148495	*R. ornithinolytica*	99
LPDA5	MW148496	*R. ornithinolytica*	99
LPDB1	MW148497	*R. ornithinolytica*	99
LPDSS1	MW148506	*R. ornithinolytica*	99
LPDSS2	MW148507	*R. ornithinolytica*	99
LPDSS3	MW148508	*R. ornithinolytica*	99
LPDSS4	MW148509	*R. ornithinolytica*	100
LPDT1	MW148510	*R. ornithinolytica*	99
LPDT2	MW148511	*R. ornithinolytica*	99
LPDB2	MW148498	*B. cereus*	100
LPDB3	MW148499	*B. cereus*	99
LPDB4	MW148500	*B. cereus*	100
LPDB5	MW148501	*B. cereus*	99
LPDS1	MW148502	*E. faecalis*	100
LPDS2	MW148503	*E. faecalis*	100
LPDS3	MW148504	*E. faecalis*	100
LPDS4	MW148505	*E. faecalis*	99

LPD=Lam Pao Dam, *R. ornithinolytica*=*Raoultella ornithinolytica*, *B. cereus*=*Bacillus cereus*, *E. faecalis*=*Enterococcus faecalis*

### Presence of resistance genes in the isolated bacteria

All the isolated bacteria were capable of growing in culture media containing ampicillin. We investigated the beta-lactamase and carbapenem genes of the bacterial isolates and found that most *R. ornithinolytica* isolates harbored *bla*_SHV_ and *bla*_OXA_. Furthermore, *R. ornithinolytica* LPDA1 and LPDA2 harbored *bla*_NDM-1_ in their plasmid DNA (Table-2). However, we could not detect resistance genes in the four *B. cereus* isolates, whereas all the four *E. faecalis* isolates harbored *bla*_IMP_ in their plasmids (Table-2). These data indicate that most of the isolated bacterial strains contain resistance genes.

**Table-2:** Resistance genes detection in 20 isolated bacterial strains.

Bacterial strains	Antibiotics resistance genes					

	*bla*_TEM_	*bla*_SHV_	*bla*_OXA_	*bla*_KPC-2_	*bla*_NDM-1_	*bla*_IMP_
*R. ornithinolytica* LPDA1	-	+	+	-	+	-
*R. ornithinolytica* LPDA2	-	+	+	-	+	-
*R. ornithinolytica* LPDA3	-	+	+	-	-	-
*R. ornithinolytica* LPDA4	-	+	+	-	-	-
*R. ornithinolytica* LPDA5	-	+	+	-	-	-
*R. ornithinolytica* LPDB1	-	+	+	-	-	-
*R. ornithinolytica* LPDSS1	-	+	+	-	-	-
*R. ornithinolytica* LPDSS2	-	+	+	-	-	-
*R. ornithinolytica* LPDSS3	-	+	+	-	-	-
*R. ornithinolytica* LPDSS4	-	+	+	-	-	-
*R. ornithinolytica* LPDT1	-	-	+	-	-	-
*R. ornithinolytica* LPDT2	-	+	+	-	-	-
*B. cereus* LPDB2	-	+	-	-	-	-
*B. cereus* LPDB3	-	-	-	-	-	-
*B. cereus* LPDB4	-	-	-	-	-	-
*B. cereus* LPDB5	-	-	-	-	-	-
*E. faecalis* LPDS1	-	-	-	-	-	+
*E. faecalis* LPDS2	-	-	-	-	-	+
*E. faecalis* LPDS3	-	-	-	-	-	+
*E. faecalis* LPDS4	-	-	-	-	-	+

LPD=Lam Pao Dam, *R. ornithinolytica*=*Raoultella ornithinolytica*, *B. cereus*=*Bacillus cereus*, *E. faecalis*=*Enterococcus faecalis*

### Analysis of antibiotic-resistant populations

The top 10 most abundant bacterial species in the LPD sample are shown in Table-3. The most abundant species in the LPD sample was *Exiguobacterium indicum*, constituting 55.7% of the total microbial community, followed by *E. faecalis* that accounted for 34.5%. The remaining 7.94% of the community was composed of a variety of other species. *Raoultella* *ornithinolytica* showed the highest abundance among the three species, comprising 0.95%. Similarly, other species were found in the ampicillin-resistant population, including *Aneurinibacillus migulanus*, *Serratia marcescens*, *Escherichia coli*, *Lysinibacillus xylanilyticus*, *Clostridium* spp. CYP8, *Paraclostridium bifermentans*, and *Bacteroides vulgatus*. The *B. cereus* population comprised <0.03% of species abundance.

**Table-3:** Top 10 relative abundances of species in Lam Pao Dam sample.

Taxonomy	Relative abundance (%)
*Exiguobacterium indicum*	55.7
*Enterococcus faecalis*	34.5
*Raoultella ornithinolytica*	0.95
*Aneurinibacillus migulanus*	0.33
*Serratia marcescens*	0.20
*Escherichia coli*	0.16
*Lysinibacillus xylanilyticus*	0.06
*Clostridium* spp. CYP8	0.06
*Paraclostridium bifermentans*	0.04
*Bacteroides vulgatus*	0.03
Others	7.94

### Antimicrobial susceptibility testing

We evaluated the MICs of 10 *R. ornithinolytica* strains against the following five antibiotics: Ampicillin, meropenem, doripenem, ertapenem, and imipenem (Table-4). All 10 *R. ornithinolytica* strains were found to be resistant to ampicillin and susceptible to meropenem, doripenem, ertapenem, and imipenem. The MICs of doripenem and ertapenem against *R. ornithinolytica* were 0.25 and 0.0625 μg/mL, respectively.

**Table-4:** MICs of isolated *R. ornithinolytica* strains.

Isolates	MIC (mg/mL)				

	Ampicillin	Meropenem	Doripenem	Ertapenem	Imipenem
*R. ornithinolytica* LPDA1	>8	0.125	0.25	0.0625	2
*R. ornithinolytica* LPDA2	>8	0.125	0.25	0.0625	2
*R. ornithinolytica* LPDA3	>8	0.25	0.25	0.0625	2
*R. ornithinolytica* LPDA4	>8	0.25	0.25	0.0625	2
*R. ornithinolytica* LPDA5	>8	0.25	0.25	0.0625	2
*R. ornithinolytica* LPDB1	>8	0.25	0.25	0.0625	2
*R. ornithinolytica* LPDSS1	>8	0.25	0.25	0.0625	2
*R. ornithinolytica* LPDSS2	>8	0.25	0.25	0.0625	2
*R. ornithinolytica* LPDSS3	>8	0.25	0.25	0.0625	4
*R. ornithinolytica* LPDSS4	>8	0.25	0.25	0.0625	4
*R. ornithinolytica* LPDT1	>8	0.25	0.25	0.0625	4
*R. ornithinolytica* LPDT2	>8	0.25	0.25	0.0625	2

LPD=Lam Pao Dam, *R. ornithinolytica*=*Raoultella ornithinolytica*, MIC=Minimum inhibitory concentration

## Discussion

Antibiotic resistance is a major health threat throughout the world. The rising prevalence of drug-resistant bacteria poses a serious challenge to the effective treatment of infections in both humans and animals. Water is a primary source of human and animal exposure to bacteria, and the presence of drug-resistant bacteria in water could present a serious threat to public health and animal production. Drug-resistant bacteria can exert significant effects on animal health. These bacteria can cause difficult-to-treat infections and can also spread antibiotic resistance to other bacteria. In some cases, drug-resistant infections can be fatal for animals [6]. This problem is an increasing concern. The increasing prevalence of these bacteria makes it more difficult to treat livestock infections, which can result in significant economic losses for farmers and ranchers.

**Figure-2:**
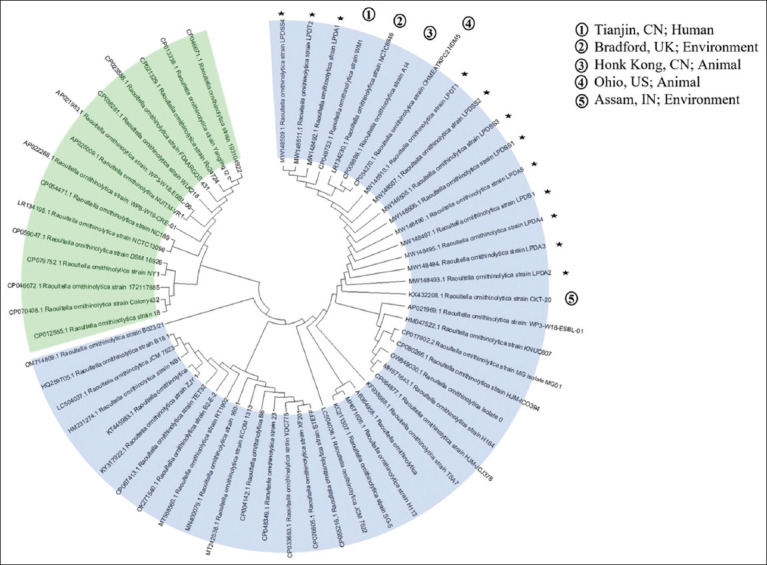
Phylogenetic analysis of the partial 16S rDNA sequences of *Raoultella ornithinolytica* strains. Stars indicate Lam Pao Dam isolates and blue and green represent two distinct clades of *R. ornithinolytica* sequences.

This study explored the presence of drug-resistant bacteria in LPD water collected from two banks near Thepsuda bridge at LPD (Figure-1), which is a significant tourist spot with cultured fish cages. Despite long-term water use, the potential risks of contamination, such as drug-resistant bacteria, have not been investigated in this dam. Our results revealed the presence of 12 ampicillin-resistant *R. ornithinolytica* isolates in the dam water. *Raoultella* *ornithinolytica* is commonly found in aquatic environments [10] and is considered a globally emerging dangerous zoonotic pathogen [11]. It has been detected in aquatic environments, including well water in China [19], wastewater in Japan [20], wastewater treatment plants in Spain [21, 22] and the US [23], seafood processing plant in Thailand [24], and river water in the Philippines [25]. Nonetheless, the phylogenetic analysis of the 16s rDNA sequence isolated from *R. ornithinolytica* strains showed similarities to the isolates from China, UK, US, and India, which were isolated from human, animal, and environmental samples (Figure-2). Furthermore, the LPD water contained four isolates each of *B. cereus* and *E. faecalis*. *Bacillus* *cereus* is a spore-forming bacterium and can cause food poisoning [26], and *E. faecalis* is a common species in the human gut and can cause infections in the urinary tract, bloodstream, and other sites [27].

The presence of 20 ampicillin-resistant bacterial isolates suggests that the isolated strains tend to harbor antibiotic-resistant genes, such as beta-lactamase and/or carbapenemase genes. Our results demonstrated that almost all *R. ornithinolytica* isolates harbored *bla*_SHV_ and *bla*_OXA_. *bla*_SHV_ is recognized as the most prevalent ESBL gene that confers antibiotic resistance to pathogenic bacteria worldwide, primarily in *E. coli* and *Klebsiella* spp. [28–31], whereas *bla*_OXA_ is typically identified in *Pseudomonas aeruginosa* [32] and *Acinetobacter baumannii* [33]. However, *R. ornithinolytica* harboring *bla*_SHV_ and *bla*_OXA_ has been isolated from a patient in China [34]. *Raoultella ornithinolytica* LPDA1 and LPDA2 strains also harbored *bla*_NDM-1_, a gene that has been detected in the plasmid of *R. ornithinolytica* isolated from human stool samples [35] and river sediments [17]. Furthermore, all four *E. faecalis* isolates contained *bla*_IMP_, a gene that has been detected in an *Enterococcus* spp. isolate from fishery lakes in Romania [36]. Our study demonstrates the presence of genes that exhibit resistance to last-resort antibiotics on mobile genetic elements in a dam in Thailand, suggesting their potential for easy spread and the dam acting as a reservoir of antibiotic resistance.

In the LPD sample, *E. indicum* was identified as the most abundant species, followed by *E. faecalis* and *R. ornithinolytica*. *Exiguobacterium* *indicum* was not isolated from the water sample, possibly because of aerobic culturing conditions. This is intriguing because *E. indicum* is a relatively rare bacterium and not commonly detected in water samples. Its presence in the LPD sample indicates that it may be more widespread in water bodies than had been previously believed. The presence of these three species in the LPD sample suggests potential contamination. Nevertheless, it is important to note that the relative abundance of each species does not necessarily indicate the severity of infection. For instance, a small number of *R. ornithinolytica* cells can cause a severe infection, whereas a large number of *E. faecalis* cells may not produce any symptoms.

The MICs of 10 *R. ornithinolytica* strains against six antibiotics revealed resistance to ampicillin but susceptibility to meropenem, doripenem, ertapenem, and imipenem, which are often used to treat infections caused by multidrug-resistant bacteria. This finding is expected because ampicillin is less effective against Gram-negative bacteria. However, it is important to note that the MICs of carbapenems against *R. ornithinolytica* strains were relatively high. For instance, the MIC of imipenem against *R. ornithinolytica* LPDSS3, LPDSS4, and LPDT1 was 4 mg/mL. Nevertheless, our study investigated only the susceptibility of *R. ornithinolytica* isolates to carbapenem drugs, which may not be adequately comprehensive. Further studies are necessary to evaluate the drug sensitivity of *R. ornithinolytica* against a wider range of antibiotics.

The presence of drug-resistant bacteria in LPD water is concerning because they may present a serious threat to human health, especially if resistant to commonly used antibiotics. Nonetheless, our study has limitations because it was conducted in a single location, which may limit its generalizability to other water bodies. Our findings emphasize the need for increased surveillance of antibiotic resistance in water, considering its significance as a major source of human exposure to bacteria, which could potentially harm public health.

## Conclusion

Drug-resistant bacteria were identified in LPD water, including 12 *R. ornithinolytica*, 4 *B. cereus*, and 4 *E. faecalis* strains. The most common drug resistance genes were *bla*_SHV_ and *bla*_OXA_, both of which are beta-lactamase genes. Two *R. ornithinolytica* isolates also harbored the carbapenemase gene *bla*_NDM-1_. *Exiguobacterium indicum* was the most abundant species in the LPD sample, followed by *E. faecalis* and *R. ornithinolytica*, indicating potential contamination. *Raoultella* *ornithinolytica* strains were resistant to ampicillin but susceptible to other antibiotics. This study highlights a high prevalence of drug-resistant bacteria in LPD water, which raises concerns about the potential spread of antibiotic-resistant infections in animals and humans, with potential effects on public health issues and animal production.

## Authors’ Contributions

CK and MT: Performed the experimental work. PW, NS, and AT: Analyzed data. MT, PW, JT, and AT: Participated in the sample collection. AT: Planned and designed the experiment and drafted and revised the manuscript. All the authors have read, reviewed, and approved the final manuscript.
